# A giant pelvic solitary fibrous tumor with Doege–Potter syndrome successfully treated with transcatheter arterial embolization followed by surgical resection: a case report

**DOI:** 10.1186/s40792-020-01076-5

**Published:** 2020-11-25

**Authors:** Kizuki Yuza, Jun Sakata, Hiroki Nagaro, Takuya Ando, Yuki Hirose, Kohei Miura, Kazuyasu Takizawa, Takashi Kobayashi, Hiroshi Ichikawa, Takaaki Hanyu, Yoshifumi Shimada, Masayuki Nagahashi, Shin-Ichi Kosugi, Toshifumi Wakai

**Affiliations:** 1 Division of Digestive and General Surgery, Niigata University Graduate School of Medical and Dental Sciences, 1-757 Asahimachi-dori, Chuo-ku, Niigata, Niigata 951-8510 Japan; 2 Department of Digestive and General Surgery, Uonuma Institute of Community Medicine, Niigata University Medical and Dental Hospital, Niigata, Japan

**Keywords:** Solitary fibrous tumor, Non-islet cell tumor hypoglycemia, Doege–Potter syndrome, Insulin-like growth factor-II, Preoperative transcatheter arterial embolization, Intestinal ischemia, Surgical resection

## Abstract

**Background:**

Solitary fibrous tumor (SFT), a mesenchymal fibroblastic tumor with a hypervascular nature, rarely develops in the pelvis. Resection of a giant SFT occupying the pelvic cavity poses an increased risk of developing massive hemorrhage during resection, although surgical resection is the most effective treatment method for this tumor to achieve a potential cure. SFT rarely develops with Doege–Potter syndrome, which is known as a paraneoplastic syndrome characterized by non-islet cell tumor hypoglycemia (NICTH) secondary to SFT that secretes insulin-like growth factor-II (IGF-II). We present a case of a giant pelvic SFT with Doege–Potter syndrome, which was successfully treated with transcatheter arterial embolization (TAE) followed by surgical resection.

**Case presentation:**

A 46-year-old woman presented with a disorder of consciousness due to refractory hypoglycemia. Images of the pelvis showed a giant and heterogeneously hypervascular mass displacing and compressing the rectum. Endocrinological evaluation revealed low serum levels of insulin and C-peptide consistent with NICTH. Angiography identified both the inferior mesenteric artery and the bilateral internal iliac artery as the main feeders of the tumor. To avoid intraoperative massive bleeding, super-selective TAE was performed for the tumor 2 days prior to surgery. Hypoglycemia disappeared after TAE. The tumor was resected completely, with no massive hemorrhage during resection. Histologically, it was diagnosed as IGF-II-secreting SFT. Partial necrosis of the rectum in the specimen was observed due to TAE. The patient was followed up for 2 years and no evidence of disease has been reported.

**Conclusions:**

Preoperative angiography followed by TAE is an exceedingly helpful method to reduce intraoperative hemorrhage when planning to resect SFT occupying the pelvic cavity. Complications related to ischemia should be kept in mind after TAE, which needs to be planned within 1 or 2 days before surgery. TAE for tumors may be an option in addition to medical and surgical treatment for persistent hypoglycemia in Doege–Potter syndrome.

## Background

Solitary fibrous tumor (SFT) is a rare mesenchymal fibroblastic tumor with a hypervascular nature, which most commonly develops in the pleura followed by the retroperitoneal and abdominopelvic cavity [1]. SFT that develops in the pelvis is sometimes discovered as a giant tumor, which poses an increased risk of developing massive hemorrhage during resection, although surgical resection is the most effective treatment method for SFTs to achieve a potential cure. This tumor rarely develops with non-islet cell tumor hypoglycemia (NICTH), which is known as a paraneoplastic syndrome caused by extra-pancreatic tumors that secrete insulin-like growth factor-II (IGF-II) [2]. This clinical condition caused by SFT is called Doege–Potter syndrome. In this case report, we present our experience of a giant pelvic SFT with Doege–Potter syndrome, which was successfully treated with transcatheter arterial embolization (TAE) followed by surgical resection.

## Case presentation

**Fig. 1:**
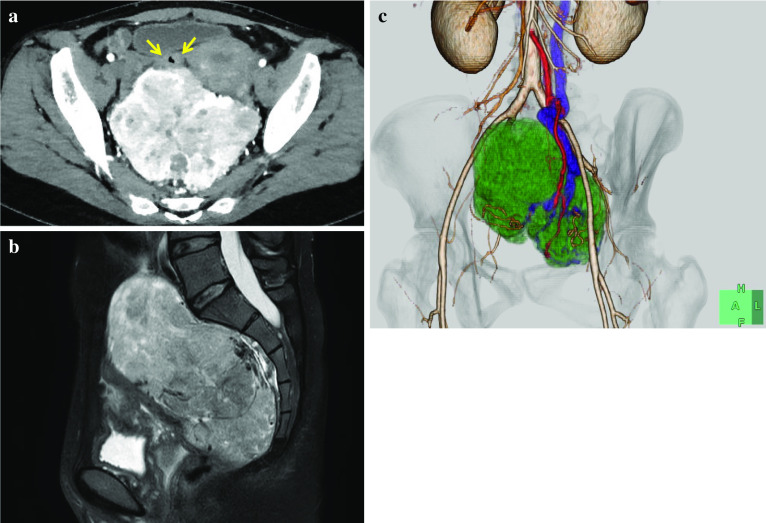
Computed tomography (CT) and magnetic resonance imaging (MRI).** a** Contrast-enhanced CT image showed a hypervascular mass occupying the pelvic cavity, displacing and compressing the rectum (yellow arrows). The tumor showed marked enhancement in a heterogeneous pattern. **b** T2-weighted pelvic MRI image showed a high-signal-intensity tumor in the pelvis. **c** 3D-CT image reconstruction showed the tumor occupying the pelvis (green), the tumor-feeding artery (the inferior mesenteric artery colored in red), and the tumor draining vein (the inferior mesenteric vein colored in blue)

A 46-year-old woman was referred to our hospital for further examination and treatment of persistent hypoglycemia and a giant pelvic mass. She had no relevant medical history, including diabetes. No evident mass was palpable in the abdomen. Laboratory examination, including tumor markers, revealed no abnormalities except for hypoglycemia and suppressed serum levels of insulin and C-peptide upon endocrinological evaluation (Table 1). Contrast-enhanced computed tomography (CT) and magnetic resonance imaging (MRI) revealed a giant hypervascular tumor occupying most of the pelvic cavity, displacing and compressing the rectum. The tumor showed marked enhancement with a heterogeneous pattern suggestive of a mesenchymal tumor (Fig. 1a, b). The inferior mesenteric artery and vein were speculated to be the feeding artery and draining vein of the tumor, respectively (Fig. 1c). Colonoscopy showed no sign of tumor invasion to the mucosa of the rectum. Based on the suppressed serum levels of insulin and C-peptide accompanying the giant pelvic mesenchymal tumor, a preoperative diagnosis of SFT with NICTH (Doege–Potter syndrome) was made. During the diagnostic work-up, intravenous hyperalimentation in addition to oral normal diet was needed to control hypoglycemia; nevertheless, she often suffered from hypoglycemia, and oral or intravenous glucose intake was needed.

**Table 1:** Endocrinological evaluation

Test	Results	Reference range
Plasma glucose (after overnight fast) (mg/dL)	36	73–109
Insulin (after overnight fast) (μU/mL)	< 1	1.1–17.0
C-peptide (after overnight fast) (ng/mL)	0.01	0.8–2.5
Hemoglobin A1c (%)	5.0	4.9–6.0
Growth hormone (ng/mL)	0.2	≤ 2.1
Prolactin (ng/mL)	20.6	4.1–28.9
ACTH (pg/mL)	14.9	7.2–63.3
LH (mIU/mL)	4.9	0.5–68.7
FSH (mIU/L)	6.7	1.5–168.8
TSH (μIU/mL)	1.34	0.50–5.00
Free T3 (pg/mL)	2.2	2.3–4.0
Free T4 (ng/dL)	0.90	0.90–1.70
Cortisol (μg/dL)	7.2	6.4–21.0
Plasma epinephrine (ng/mL)	0.03	≤ 0.1
Plasma norepinephrine (ng/mL)	0.10	0.1–0.5
Plasma dopamine (ng/mL)	< 0.01	0.82.5

Suppressed levels of insulin and C-peptide were observed, suggesting a diagnosis of non-islet cell tumor hypoglycemia

*ACTH* adrenocorticotropic hormone, *LH* luteinizing hormone, *FST* follicle-stimulating hormone, *TSH* thyroid-stimulating hormone

**Fig. 2:**
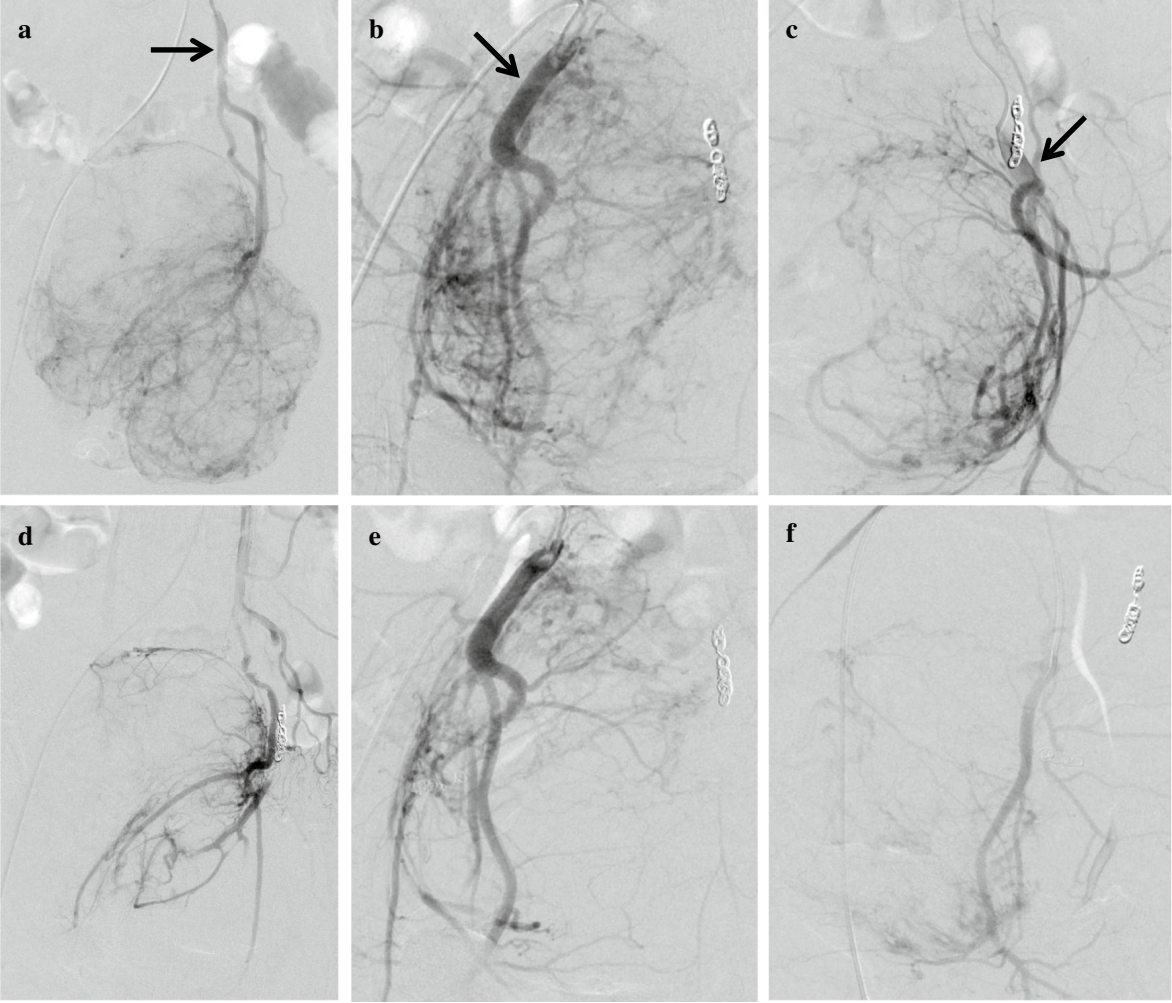
Preoperative angiography and transcatheter arterial embolization. Angiography revealed that the tumor was mainly supplied by the peripheral branches of the inferior mesenteric artery (**a**, arrow) and both right (**b**, arrow) and left internal iliac artery (**c**, arrow). Super-selective catheterization and embolization of these vessels were performed using coil and gel-foam particles resulting in reduced tumor staining (**d**–**f**)

**Fig. 3:**
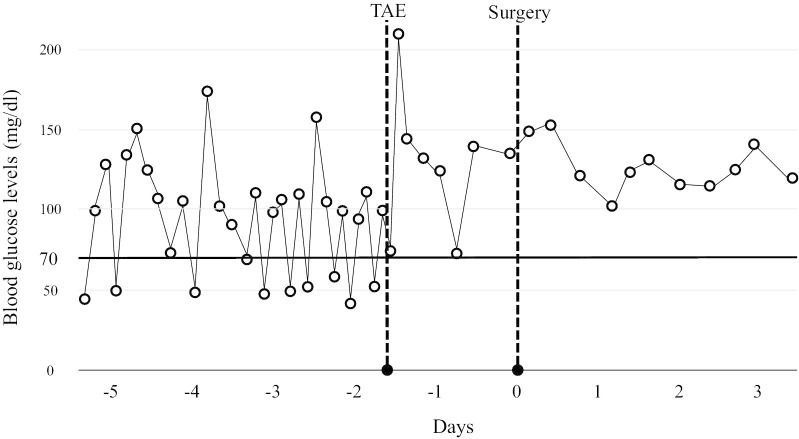
Perioperative blood glucose levels of the patient. The patient frequently suffered from hypoglycemia with blood glucose level measuring under 70 mg/dL even with hyperalimentation in addition to normal ingestion, and oral or intravenous glucose intake was needed. However, after the transcatheter arterial embolization (TAE) and surgery, she did not have hypoglycemia, and the blood glucose level stabilized

To prevent excessive surgical hemorrhage, preoperative TAE was scheduled 2 days before surgery. Angiography revealed that the tumor was supplied by branches arising from both the inferior mesenteric artery and the bilateral internal iliac artery. Super-selective catheterization and embolization of these vessels were performed using coil and gel-foam particles (Fig. 2). After the TAE, blood glucose level had stabilized, and no intravenous administration of glucose was needed (Fig. 3). On the other hand, the patient had fever and lower abdominal pain, which was controlled by oral analgesics. Ingestion was stopped, and preoperative mechanical bowel preparation was omitted. The fever had disappeared before surgery, and as scheduled, the tumor was safely resected with the rectum (low anterior resection with diverting ileostomy) 2 days after TAE. The operative time was 308 min, and the estimated blood loss was 335 mL, without the need for a blood transfusion. Considering the tumor’s size and hypervascular nature, we chose open laparotomy over a laparoscopic approach.

**Fig. 4:**
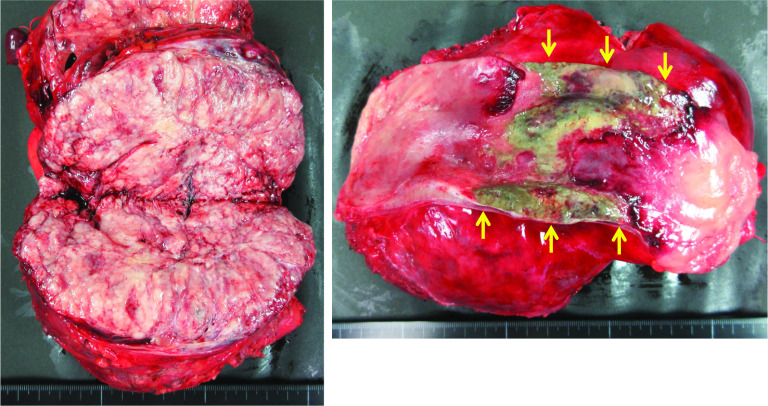
Macroscopic appearance of the tumor. The cut surface was yellowish-white in color and elastic-soft. Partial necrosis of the rectum was evident as a result of preoperative transcatheter arterial embolization (yellow arrows)

**Fig. 5:**
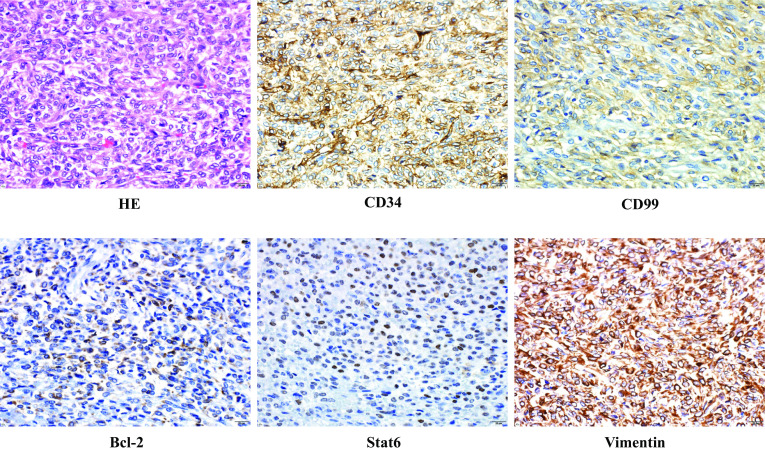
Pathological findings. Hematoxylin and eosin staining of the tumor showed monotonous spindle cell proliferation without atypia. The mitosis rate was low (2/10 high-power fields). Immunohistochemical staining demonstrated that the tumor cells were positive for CD34, CD99, Bcl-2, STAT6, and vimentin, consistent with a diagnosis of solitary fibrous tumor

**Fig. 6:**
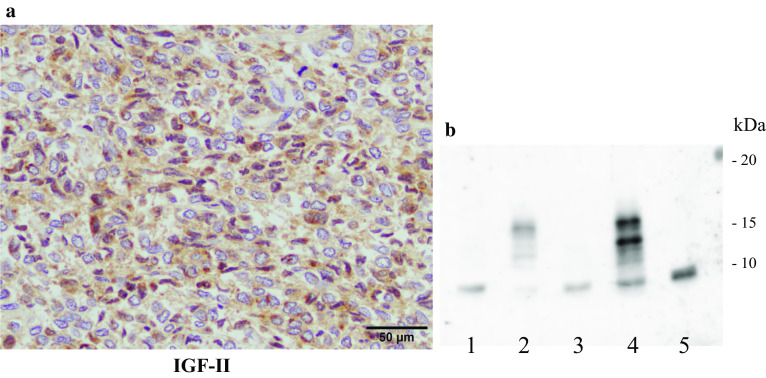
Immunohistochemical staining of insulin-like growth factor-II (IGF-II) and western blotting of high-molecular-weight IGF-II. **a** The tumor cells were positive for IGF-II staining. **b** Lane 1, serum collected from the patient on postoperative day 5; lane 2, serum collected from the patient preoperatively; lane 3, serum collected from a healthy control; lane 4, serum collected from a different patient diagnosed as IGF-II dependent non-islet cell tumor induced hypoglycemia as a positive control of high-molecular-weight IGF-II; lane 5, standard IGF-II (7.5 kDa). Large amounts of high-molecular-weight IGF-II (approximately 16 kDa) were detected in the serum collected preoperatively (lane 2) but not in serum collected postoperatively (lane 1)

The tumor measured 17 cm in diameter and weighed 850 g. The tumor size had not reduced following embolization. The cut surface was yellowish-white in color and elastic-soft. A part of the rectum wall showed necrosis because of preoperative TAE (Fig. 4). Histologically, the tumor consisted of monotonous spindle cell proliferation without atypia. The mitosis rate was low (2/10 high-power fields). Immunohistochemical staining demonstrated that the tumor cells were positive for CD34, CD99, Bcl-2, STAT6, and vimentin, consistent with a diagnosis of SFT (Fig. 5). The tumor was also positive for IGF-II staining, and we confirmed by western blotting that the circulating high-molecular-weight IGF-II had decreased after tumor removal, concomitant with remission of hypoglycemia (Fig. 6). The patient had an uneventful postoperative course. Six months later, she underwent ileostomy closure. No evidence of recurrence has been reported in the 2-year follow-up after the initial surgery.

## Discussion

In this case report, we present two important clinical implications. First, preoperative angiography followed by TAE enables safe and complete resection of SFTs occupying the narrow pelvic cavity, although careful management to avoid complications related to intestinal ischemia is needed. Second, embolization of the tumor-feeding artery may be an effective option in addition to medical and surgical treatment for persistent hypoglycemia due to Doege–Potter syndrome.

Although surgical resection is the definitive treatment in most cases of SFTs, large-sized tumors with a hypervascular nature that occupy the pelvic cavity frequently make surgical removal technically difficult [3]. Previous reports have revealed that surgical resection of SFTs occupying the pelvic cavity can be dangerous, associated with a large amount of intraoperative hemorrhage up to 13,660 mL [4–6], and may even result in intraoperative death due to uncontrollable hemorrhage [3]. Moreover, such giant tumors make it difficult for surgeons to presume their feeding arteries from preoperative imaging because of the displacement and compression of the adjacent organs by the tumor. In this context, precise preoperative identification of tumor-feeding vessels using angiography followed by TAE is a prerequisite for achieving complete resection without unexpected intraoperative hemorrhage for such giant pelvic SFTs. In our case, CT and MRI showed apparent signs of the inferior mesenteric artery as the main feeding artery of the SFT, and the involvement of the iliac artery was not suspected. However, angiography showed that the tumor was also supplied by the bilateral internal iliac artery, which led to a precise understanding of the hemodynamics of the tumor, along with safe and complete resection after TAE. Surgeons should consider preoperative angiography and TAE when planning resection of hypervascular tumors, such as SFTs, especially large tumors in the pelvic cavity, in order to achieve satisfactory results.

When performing TAE for SFTs, we should be aware of complications related to ischemia. Some authors have described the use of preoperative TAE with no complications in abdominopelvic SFTs irrigated by the iliac artery [7, 8]. In our patient, the tumor was also supplied from the bilateral iliac artery, but the dominant vascularity was the inferior mesenteric artery. Since we were concerned about intestinal ischemia after embolization, we scheduled TAE 2 days prior to the surgery. Despite performing super-selective arterial embolization of the tumor with meticulous attention not to embolize the rectum and the sigmoid colon, the patient suffered from abdominal pain and fever, and operative findings revealed partial necrosis of the rectum. There are no guidelines that reveal when to perform preoperative TAE to reduce the amount of intraoperative hemorrhage. Considering the potential of intestinal ischemia, especially for tumors in which the inferior mesenteric artery is the main feeder, it is reasonable to perform such TAE within 1 or 2 days before surgery, and the patient should be carefully observed after TAE to avoid overlooking the symptoms of ischemic complications.

NICTH is most commonly described with tumors of mesenchymal or hepatic origin. Of the 288 NICTH cases reviewed, 22% were SFTs that were commonly located in the pleura, retroperitoneum, abdomen, and pelvis [9]. Initial management of hypoglycemia in NICTH involves increased caloric intake and frequent intravenous administration of glucose or dextrose. Total resection of the tumor is curative for hypoglycemia in many cases. For cases of uncontrollable hypoglycemia or unresectable tumors, glucocorticoid administration and local antitumor therapy, such as systemic chemotherapy [10], molecular targeted therapy [11], and radiation therapy [12] have been reported to be successful in resolving hypoglycemia. TAE for tumors was effective in treating NICTH in a hepatic fibrosarcoma case [13] but not in four cases of SFT [14]. To the best of our knowledge, this is the first case that showed TAE for a tumor to be an effective method for controlling hypoglycemia in Doege–Potter syndrome. For Doege–Potter syndrome with refractory hypoglycemia or inoperable tumors, TAE may be an option for resolving hypoglycemia.

## Conclusions

Preoperative angiography followed by TAE enables safe and complete resection of SFTs occupying the pelvic cavity, although careful management to avoid complications related to ischemia is needed. TAE for tumors may be an option in addition to medical and surgical treatment for persistent hypoglycemia in Doege–Potter syndrome.

## Data Availability

Data sharing is not applicable to this article as no datasets were generated or analyzed during the current study.

## Abbreviations

NICTH: Non-islet cell tumor hypoglycemia
IGF-II: Insulin-like growth factor-II
SFT: Solitary fibrous tumor
TAE: Transcatheter arterial embolization
CT: Computed tomography
MRI: Magnetic resonance imaging
